# The integrated stress response promotes B7H6 expression

**DOI:** 10.1007/s00109-019-01859-w

**Published:** 2019-12-14

**Authors:** Akram Obiedat, Yoav Charpak-Amikam, Julie Tai-Schmiedel, Einat Seidel, Mohamed Mahameed, Tony Avril, Noam Stern-Ginossar, Lorraine Springuel, Jennifer Bolsée, David E. Gilham, Priya Dipta, Miriam Shmuel, Eric Chevet, Ofer Mandelboim, Boaz Tirosh

**Affiliations:** 1 Institute for Drug Research, School of Pharmacy, Faculty of Medicine, The Hebrew University of Jerusalem, PO Box 12065, 91120 Jerusalem, Israel; 2 The Lautenberg Center for Immunology and Cancer Research, The Biomedical Research Institute Israel-Canada of the Faculty of Medicine, The Hebrew University Hadassah Medical School, Jerusalem, Israel; 3 Department of Molecular Genetics, Weizmann Institute of Science, Rehovot, Israel; 4 Inserm U1242, University of Rennes, Rennes, France; 5 Centre de lutte contre le cancer Eugène Marquis, Rennes, France; 6 Department of Research & Development, Celyad SA, Mont-Saint-Guibert, Belgium

**Keywords:** B7H6, UPR, PERK, CAR-T

## Abstract

**Abstract:**

The B7 family member, B7H6, is a ligand for the natural killer cell receptor NKp30. B7H6 is hardly expressed on normal tissues, but undergoes upregulation on different types of tumors, implicating it as an attractive target for cancer immunotherapy. The molecular mechanisms that control B7H6 expression are poorly understood. We report that in contrast to other NK cell ligands, endoplasmic reticulum (ER) stress upregulates B7H6 mRNA levels and surface expression. B7H6 induction by ER stress requires protein kinase R-like ER kinase (PERK), one of the three canonical sensors of the unfolded protein response. PERK phosphorylates eIF2α, which regulates protein synthesis and gene expression. Because eIF2α is phosphorylated by several kinases following different stress conditions, the program downstream to eIF2α phosphorylation is called the integrated stress response (ISR). Several drugs were reported to promote the ISR. Nelfinavir and lopinavir, two clinically approved HIV protease inhibitors, promote eIF2α phosphorylation by different mechanisms. We show that nelfinavir and lopinavir sustainably instigate B7H6 expression at their pharmacologically relevant concentrations. As such, ER stress and ISR conditions sensitize melanoma targets to CAR-T cells directed against B7H6. Our study highlights a novel mechanism to induce B7H6 expression and suggests a pharmacological approach to improve B7H6-directed immunotherapy.

**Key messages:**

- B7H6 is induced by ER stress in a PERK-dependent mechanism.
- Induction of B7H6 is obtained pharmacologically by HIV protease inhibitors.
- Exposure of tumor cells to the HIV protease inhibitor nelfinavir improves the recognition by B7H6-directed CAR-T.

**Electronic supplementary material:**

The online version of this article (10.1007/s00109-019-01859-w) contains supplementary material, which is available to authorized users.

## Introduction

Natural killer (NK) cells are effector lymphocytes of the innate immune system. They are able to recognize and lyse tumor and virus-infected cells without priming. Their activity is governed by a delicate balance between activating and inhibitory receptors. Among the activating receptors are the natural cytotoxicity receptors (NCRs), a family of three members: NKp30, NKp44, and NKp46. These receptors bind a diverse array of cellular- and pathogen-associated ligands, and they induce the killing activity of NK cells [1, 2].

B7 homolog 6 (B7H6), also known as NCR3LG1 (natural killer cell cytotoxicity receptor 3 ligand 1), is a human-specific B7 family member that binds to and activates the NKp30 receptor. B7H6 is typically not expressed on normal human tissues, but it is expressed on approximately 20% of human tumor cell lines including melanoma, carcinomas, T and B lymphomas, and myeloid leukemias, as well as primary tumor blood and bone marrow cells [3, 4]. Besides its expression in cancer, B7H6 is upregulated under inflammatory conditions such as atopic dermatitis [5], and it is induced upon stimulation by ligands of toll-like receptors or proinflammatory cytokines at the surface of proinflammatory monocytes and neutrophils [6]. B7H6 is also important for fighting viral infections. Some viruses, such as the human cytomegalovirus (HCMV) and the human herpesvirus 6, evolve mechanisms to downregulate B7H6, a strategy that probably helps to escape immune detection [7–9]. Other viruses not equipped with these machineries, such as the human immunodeficiency virus 2 (HIV-2), confer the upregulation of B7H6 [10]. The wide expression profile on tumors and the lack of expression on healthy tissues highlight B7H6 as a promising target for immunotherapies. B7H6-specific chimeric antigen receptor (CAR) T cells and B7H6-specific bispecific T cell engagers (BiTEs) show a potent antitumor activity in vitro and in vivo [11, 12]. While being a potential target for cancer immunotherapy, the mechanisms that control B7H6 expression in tumors are poorly understood. It was recently reported that B7H6 transcription is regulated via the proto-oncogene Myc in a variety of tumor cells [13] and by the long non-coding RNA LINC00673 in breast cancer [14]. However, these mechanisms cannot account for the entire expression pattern of B7H6 in tumors.

The endoplasmic reticulum (ER) is the first compartment of the secretory pathway, in which proteins destined to other organelles are folded and integrated into the membrane. Conditions of ER stress develop following the accumulation of improperly or partially folded proteins in the ER or other perturbations to ER homeostasis. Signaling pathways are activated upon ER stress in an attempt to restore ER homeostasis, collectively termed the unfolded protein response (UPR). The UPR is triggered by three key transmembrane proteins: inositol-requiring enzyme 1 (IRE1), protein kinase R (PKR)-like endoplasmic reticulum kinase (PERK), and activating transcription factor 6 (ATF6). Upon accumulation of improperly folded proteins in the ER, IRE1 and PERK are activated by oligomerization and trans-autophosphorylation. IRE1 contributes to the unconventional splicing of the mRNA of the transcription factor X-box binding protein 1 (XBP1) to produce its active and more stable form of spliced XBP1 (XBP1s), which activates the transcription of UPR target genes [15]. PERK is activated similarly to IRE1 and inhibits protein translation by phosphorylation of the eukaryotic translation initiation factor 2 alpha (eIF2α), reducing the load of newly synthesized proteins entering the ER. In addition to global protein translation suppression, elevated eIF2α phosphorylation favors the translation of mRNA molecules that contain inhibitory upstream open reading frames (uORFs). This feature is conserved in eukaryotes and is shared with other stress responses, collectively termed an integrated stress response (ISR) [16, 17]. A bona fide target of the ISR in mammalian cells is an activating transcription factor 4 (ATF4) [18]. Thus, the ISR also leads to a transcription response, mediated in part by ATF4 [19].

The UPR plays important roles in the development and function of the immune system [20, 21]. It also develops in certain tumors affecting their growth and sensitivity to drugs [22]. The UPR is involved as well in immune cell recognition. For instance, the UPR plays a role in the regulation of the expression of stress-induced ligands of natural killer cells, MICA, and B (major histocompatibility complex class I polypeptide-related sequence A and B) and ULBPs (UL16 binding proteins) [23, 24]. B7H6 is also a stress-induced NK cell ligand, though its regulation by the UPR has not been reported [4]. These data prompted us to investigate the role of the UPR in B7H6 expression. We now report that of the stress-induced ligands, B7H6 is the only NK cell ligand upregulated by ER stress. This induction was dependent on PERK. Based on these observations, we tested two clinically approved anti-viral drugs, nelfinavir and lopinavir, that promote eIF2α phosphorylation albeit by different mechanisms. Nelfinavir and lopinavir, similarly to ER stress conditions, strongly and persistently induced B7H6 expression on melanoma cells. This improved the activation of B7H6-specific CAR-T cells.

## Materials and methods

### Cell lines and culturing conditions

The human melanoma cell lines 624 and 526 were provided by Dr. Michal Lotem (Hadassah Medical Center, Jerusalem, Israel). Cells were cultured in Dulbecco’s modified Eagle medium (DMEM, Sigma) supplemented with 10% fetal bovine serum (FBS, Invitrogen), 2 mM L-glutamine (Biological industries), and 1% penicillin-streptomycin and sodium pyruvate solutions (Biological industries) at 37 °C under 5% CO_2_.

### B7H6 CAR T cells

Human peripheral blood mononuclear cells (PBMCs) were isolated from whole blood or leukapheresis of healthy donors by a ficoll density gradient and activated for 2 days with OKT-3 (Miltenyi) in the presence of IL-2 (Miltenyi). Activated cells were then transduced with a retroviral vector coding for the anti-B7H6 CAR fused to a Furin T2A cleaving site in frame with a truncated human CD19 marker (tCD19) described in [11] or a vector coding for the truncated CD19 vector described in [11]. Two days later, transduced T cells were enriched using CD19-specific magnetic beads (Miltenyi, 130-050-301), and then expanded for an additional 4 days together with IL-2. At day 8, harvested cells were evaluated for cell number and viability prior to cryopreservation. Surface expression of the CAR on T cells was validated by flow cytometry using recombinant human Fc-tagged B7H6 proteins and a secondary antibody recognizing the Fc part of human IgG. All human studies have been approved by the appropriate ethics committee.

### Chemicals and reagents

Thapsigargin (Tg, abcam ab120286), GSK2606414 (GSK, TOCRIS 5107), cycloheximide (CHX, Sigma-Aldrich 66819), ISRIB (Sigma-Aldrich, SML0843), nelfinavir (Nel, Glentham Life Science, GP7332), lopinavir (Lop, Sigma-Aldrich, SML1222), ionomycin (Sigma-Aldrich, I3909), PMA (Sigma-Aldrich, P1585). Stock solutions of Tg, GSK, ISRIB, Nel, and Lop were prepared in DMSO at concentrations of 2.5 mg/ml, 1 mM, 0.5 mM, 5 mM, and 10 mM, respectively. CHX was dissolved in ethanol at 50 mg/ml concentration. The final concentration of each compound was 0.125 μg/ml, 1 μM, 0.5 μM, 10 μM, 20 μM, and 100 μg/ml, respectively. The corresponding solvent was used as a vehicle control for each treatment.

### Generation of UPR-deficient cells by CRISPR/Cas9 editing

Knockout cells were produced as previously described [24]. In brief, cells were transfected with constructs targeting IRE1, PERK, or CHOP. Then, single-cell clones were generated by limiting dilution. The following sequences were used as sgRNA: IRE1 FW: 5′Phos-CACCGCTTTTATGTCTGGCAGCGGG-′3, REV: 5′Phos-AAACCCCGCTGCCAGACATAAAAGC-′3; PERK FW: 5′Phos-CACCGCCGAGGCTCCTGCTCTCCCG-′3, REV: 5′Phos-AAACCGGGAGAGCAGGAGCCTCGGC-′3; CHOP FW: 5′Phos-CACCGAGTCATTGCCTTTCTCCTTC-3′, REV: 5′Phos-AAACGAAGGAGAAAGGCAATGACTC-3′. B7H6 FW: 5′Phos-CACCGAGAGTGGGGACGCCTCACTG-3′, REV: 5′Phos-AAACCAGTGAGGCGTCCCCACTCTC-3′.

### Flow cytometry

Melanoma 624 wt or knockout cells were plated at equal densities and incubated overnight. Resuspended cells were incubated on ice for 1 h with the primary antibody at a concentration of 0.2 μg/well in FACS buffer (1× PBS, 0.5% bovine serum albumin, 0.05% NaN_3_). The cells were then incubated for 30 min on ice with anti-mouse AlexaFluor 647 secondary antibody (Jackson ImmunoResearch). The following primary antibodies were used: anti-MICA (clone 159227, R&D Systems), anti-MICB (clone 236511, R&D Systems), anti-ULBP1 (clone 170818, R&D Systems), anti-ULBP2/5/6 (clone 165903, R&D Systems), anti-ULBP3 (clone 166514, R&D Systems), anti-B7H6 (clone 875001, R&D systems), anti-PVR (in-house developed), anti-HLA1 (W6/32), anti-Beta-2 microglobulin (β2M, clone 2M2, Biolegend), anti-Ceacam-1 (clone ASL-32, Biolegend), anti-Nectin-2 (clone TX31, Biolegend). Mouse IgG1 (clone MOPC-21, Biolegend), IgG2a (clone MOPC-173, Biolegend), and IgG2b (clone MPC-11, Biolegend) were used as an isotype control.

### Generation of B7H6 5′-UTR reporters

The 5′-UTR of B7H6 upstream to GFP coding sequence (5′BG) was generated by de novo total synthesis (Syntezza Bioscience, Jerusalem, Israel) and cloned into a lentiviral vector. Then, using site-directed mutagenesis, a T in the upstream ATG of the 5′UTR was replaced by A (AAG-5′BG) (Fig. 2a). A PCR reaction was performed using Phusion DNA polymers (New England Biolabs, M0530S); then, the products were digested with DpnI (ThermoFisher, ER1701) for 1 h at 37 °C, followed by transformation and later DNA extraction. The mutation was validated by sequencing. Vectors were co-transduced into 624 wt cells. Then, 48 h post-infection, they were sorted for GFP-positive cells. A GFP vector was used as a negative control. The sorted cells were treated with 0.125 μg/ml of Tg for 16 h, and then, they were analyzed for GFP level using flow cytometry. The following primers were used for the site-directed mutagenesis: FW: 5′-GTGGGAAGTGCAAAAGCGCCGGCTGG-′3, REV: 5′-CTTTTGCACTTCCCACTTCTTCAGATCCCTTC-′3.

### Polysome profiling

Polysome profile analysis was carried out as described above [25]. Briefly, 624 wt and PERK KO cells were cultured in 10-cm dishes and treated with Tg, Nel, or Lop for 16 h. Then, they were treated with 100 μg/ml CHX for 1 min and washed twice with cold PBS containing 100 μg/ml CHX. The cells were collected and lysed with 250-μl lysis buffer (12.5 mM Tris pH = 7, 12.5 mM Tris pH = 8, 150 mM NaCl, 5 mM MgCl_2_, 1 mM dithiothreitol) supplemented with 1% triton, 30 U/ml Turbo DNase (Ambion), and 100 μg/ml cycloheximide in DEPC water. The lysed samples were centrifuged at 12,000*g* at 4 °C for 10 min. The cleared lysates were loaded onto a 10–50% sucrose gradient and centrifuged at 35,000 rpm in an SW41 rotor for 3 h at 4 °C. Gradients were fractionated into 12 fractions, and the optical density at 254 nm was continuously recorded using a Biocomp gradient station. The fractions were combined into three phases, polysome free, light, and heavy depending on the UV reading. The amount of B7H6 mRNA was determined in each phase by qRT-PCR.

### CAR T cell potency assay

Human melanoma 624 wt cells were plated and treated with Tg, Nel, and Lop for 16 h. Then, the drugs were washed and 10^5^ cells of each treatment were collected and cocultured with B7H6-specific CAR T cells in round-bottom 96-well plates at a ratio of 1:1. Supernatants were collected after 24 h and assayed for IFNγ by ELISA using DuoSet ELISA kit (R&D Systems) and LDH release (Pierce LDH Cytotoxicity Assay Kit, ThermoFisher) according to the manufacturer’s instructions.

### Quantitative PCR

Total RNA was isolated using TRI-reagent (Bio-Rad). Total RNA (1 μg) was reverse transcribed with an iScript cDNA synthesis kit (Bio-Rad) according to the manufacturer’s instructions. Quantitative PCR was used to measure mRNA expression as follows: cDNA was mixed with 0.2 μM of both the forward and reverse primers in a final volume of 5 μl and mixed with 5 μl of iTaq universal SYBR Green Supermix (Bio-Rad). hRPLP0 was used as endogenous reference gene for PCR quantification. PCR was performed on CFX Connect™ Real-Time PCR Detection System (Bio-Rad). For polysome profiling, the combined phases were treated with 8 M guanidine hydrochloride and 1 mL of 100% cold ethanol, then incubated in − 20 °C overnight. The samples were spanned down at 20,000 g for 30 min at 4 °C, washed with 75% cold ethanol, and resuspended with 1 ml Trizol; then, RNA was extracted as mentioned above. The following primers were used: RPLP0 FW: 5′-CCAACTACTTCCTTAAGATCATCCAACTA-′3, REV: 5′-ACATGCGGATCTGCTGCA-′3; B7H6 FW: 5′-TCACCAAGAGGCATTCCGAC-′3, REV: 5′-TGGGGAAGCCACAACTTCAA-′3. ATF4 FW: 5′-ATGACCGAAATGAGCTTCCTG-′3, REV: 5′-GCTGGAGAACCCATGAGGT-′3. Primers’ quantitative efficiency was validated using standard curves.

### Western blotting

Cells were plated in equal densities, whenever needed. They were treated with 0.125 μg/ml of thapsigargin, 10 μM nelfinavir, or 20 μM lopinavir for the indicated time. Cells were then lysed using RIPA buffer (25 mM Tris-HCl pH 7.6, 150 mM NaCl, 1% NP-40, 1% sodium deoxycholate, 0.1% SDS) and analyzed by SDS-PAGE. Quantification of blots was performed with the Image Lab software. The following primary antibodies were used: B7H6 (Clone EPR21841, Abcam), ATF4 (Clone D4B8, Cell Signaling), Flag (Clone M2, Sigma F1804), p-eIF2α (Clone D9G8, Cell Signaling), total eIF2α (Clone D7D3, Cell Signaling), β-actin (clone AC-15, Abcam), α-tubulin (DM1A, Abcam), p97 (polyclonal antibody was provided by Dr. Ariel Stanhil, The Open University, Israel). HRP-conjugated secondary antibodies (Goat anti-rabbit and Rabbit anti-mouse) were purchased from Jackson ImmunoResearch.

### ATF4 and B7H6 overexpression

A total of 624 wt cells were transfected using TransIT®-2020 (Mirus) reagent with Flag-ATF4 vector or transduced with Flag-B7H6 lentiviral vector. Forty-eight hours post transfection, cells were harvested and tested for Flag, ATF4, and B7H6 expression by immunoblotting.

### HCMV infection

The virus used in HCMV infection experiments is an HCMV TB40/e_GFP mutant strain deleted for the genomic region encompassing US17-20. The Virus was generated and grown as previously described [8]. For the infection, 50,000 cells were grown overnight in a 24-well plate. Next, a virus sample or only growth medium (in case of the mock-infected cells) was added and infection was amplified by centrifugation of the infected cells (800*g*, 30 min, 30 °C). The infected cells were incubated under normal conditions (5% CO_2_, 37 °C) for 48 h post-infection, then harvested and taken for analysis using flow cytometry. The presented analysis is gated only on the infected (GFP-expressing) cells.

### Statistical analysis

We applied a non-parametric Mann–Whitney *U* test or Kruskal–Wallis one-way analysis of variance to determine statistical significance at *p < 0.05.

## Results

### ER stress upregulates B7H6 in a PERK-dependent manner

**Fig. 1:**
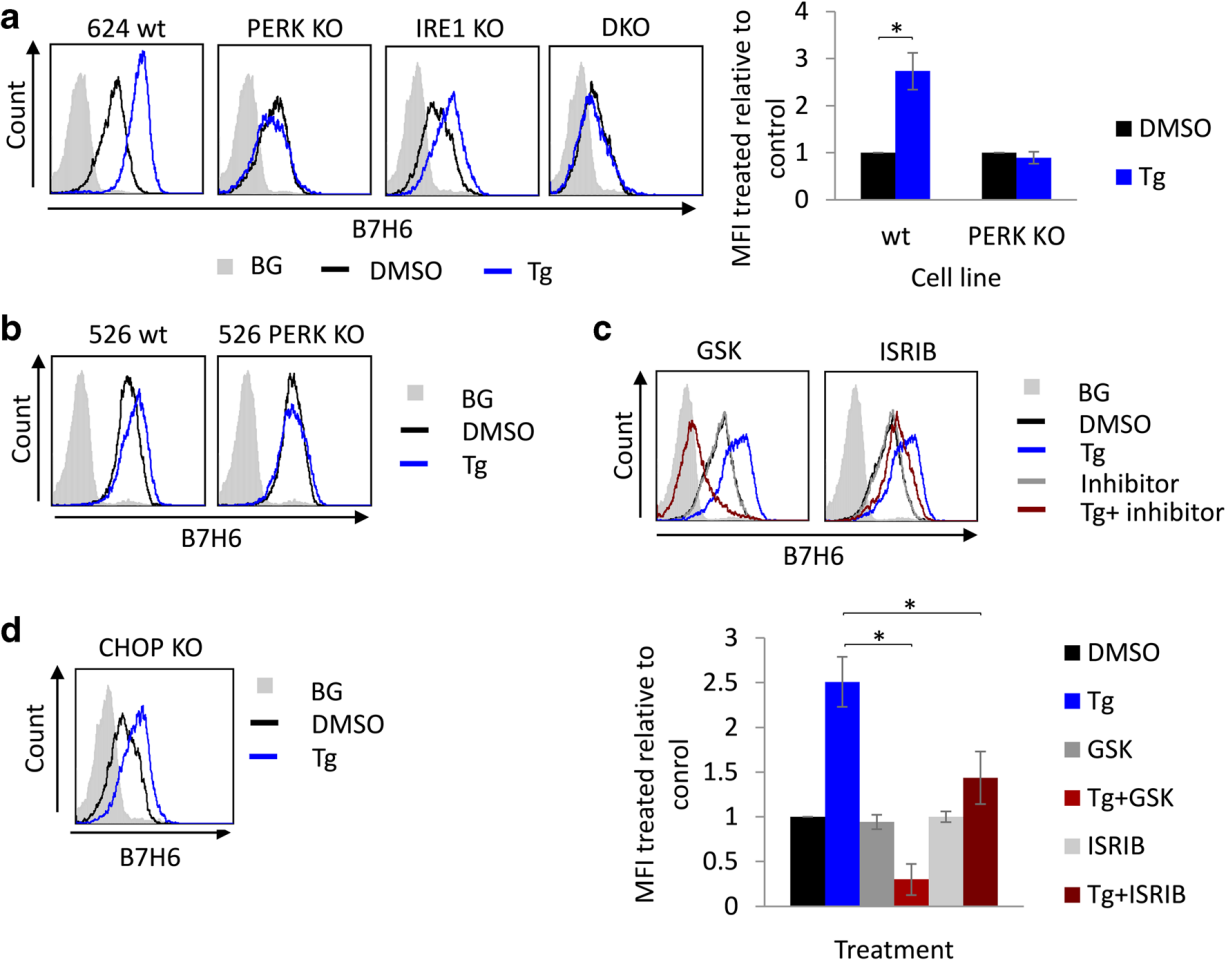
PERK is required for B7H6 upregulation under ER stress conditions. B7H6 surface levels were assessed by flow cytometry after treatment with 0.125 μg/ml thapsigargin (Tg) or mock treated with DMSO for 16 h in the following conditions: **a** 624 wt, PERK knockout (KO), IRE1 KO and PERK/IRE1 double KO (DKO) cells, to the right appears quantification of the average mean fluorescence intensity (MFI) ± STD of treated relative to untreated cells of three independent experiments. **b** Melanoma 526 wt and PERK KO cells. **c** 624 wt cells pretreated with 1 μM GSK or 0.5 μM ISRIB for 1 h. The lower panel shows quantification of the average MFI ± STD of treated relative to untreated cells of three independent experiments. **d** 624 CHOP KO cells. BG indicates secondary only background staining, which was similar for both treated and untreated cells (shown is the BG for untreated cells)

The UPR through the IRE1 arm suppresses the transcription of MICA and MICB [24]. To characterize the comprehensive effect of ER stress on the expression of NK cell ligands, we analyzed their expression on melanoma 624 cells after treatment with the ER stress inducer thapsigargin (Tg). While most of the tested ligands were neither affected nor decreased, B7H6 was the only ligand whose expression noticeably increased at the cell surface (Fig. S1). This was unexpected, as protein trafficking is usually perturbed by agents that non-discriminatorily damage protein folding in the ER, such as Tg. We used a panel of IRE1 or PERK-deficient melanoma 624 cells (described in [24]) to examine which pathways of the UPR are involved in the elevation of B7H6 expression. B7H6 surface expression was increased on IRE1 KO cells upon Tg treatment similarly to wt cells. In contrast, in cells deleted for PERK or both PERK and IRE1 (DKO, generated independently, see Fig. S2A), B7H6 expression was not affected by Tg (Fig. 1a). To ensure that the effect was not specific to these particular clones and it represents a general regulation of B7H6 by the PERK pathway, two additional PERK KO clones were tested and showed the same phenotype (Fig. S2B). In addition, we generated PERK KO in melanoma 526 cells, a melanoma cell line derived from a different patient. In this cell line, B7H6 was also induced by Tg in a PERK-dependent manner (Fig. 1b). These data indicate that ER stress promotes B7H6 expression by engaging the PERK signaling pathway.

Detection of B7H6 by immunoblotting is problematic. The commercial antibody used to detect B7H6 by flow cytometry does not recognize the protein in Western blotting applications, and the few antibodies used for B7H6 immunoblotting suffer from non-specific interactions. We were therefore concerned that the increase in B7H6 surface staining may be a technical artifact. For this purpose and for later experiments, as detailed below, B7H6 KO cells were prepared. Single-cell clones were screened with the best antibody we could obtain for Western. This effort identified a KO clone, for which the lack of surface B7H6 was validated by the flow cytometry antibody (Fig. S2C). This reassured that B7H6 is increased by ER stress in a PERK-dependent manner.

To further confirm the role of the PERK pathway in B7H6 regulation, we interfered with its signaling cascade with two drugs, each targeting the pathway at a distinct step. GSK2606414, directly inhibits PERK [26] and ISRIB, a molecule that inhibits the pathway downstream to eIF2α phosphorylation [27]. Both inhibitors reduced the upregulation of B7H6 after Tg treatment. We observed that the inclusion of the PERK inhibitor reduced even the surface expression below the untreated control (Fig. 1c). This may be a consequence of misfolding of B7H6 itself, as Tg plus GSK2606414 was shown to severely perturb ER morphology and function. This was not observed for ISRIB [28]. CHOP, which is activated downstream of PERK/eIF2α, regulated the expression of the NK cell ligand ULBP1 [23]. Using CHOP KO cells, we show that B7H6 is induced irrespectively of it (Fig. 1d). This demonstrates that B7H6 is upregulated under conditions of ER stress by the PERK pathway, most likely in a manner that depends on the phosphorylation of eIF2α.

### HCMV upregulates B7H6 in a PERK-dependent manner

**Fig. 2:**
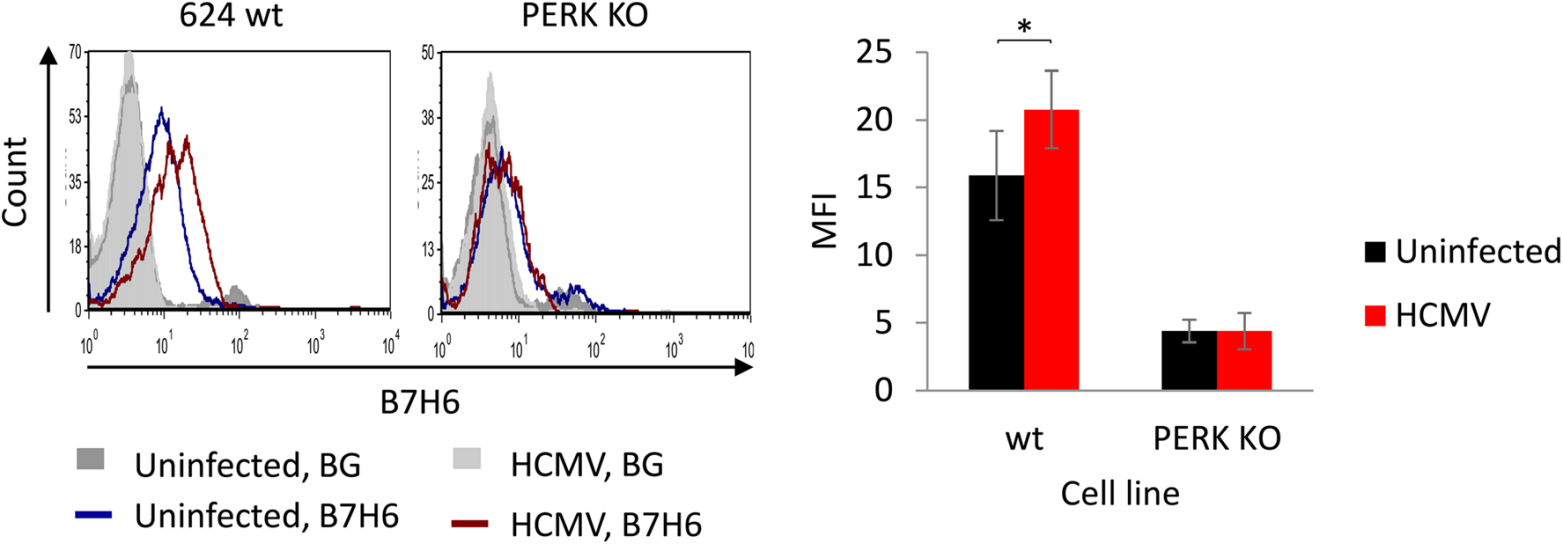
ER stress induced by HCMV is sufficient to upregulate B7H6 in a PERK-dependent manner. B7H6 surface levels were evaluated by flow cytometry on 624 wt and PERK KO cells infected with HCMV ΔUS17-20 for 48 h. The bars represent average MFI ± SEM of three independent experiments. BG indicates secondary only background staining

To examine whether physiological ER stress also results in B7H6 induction, we infected the melanoma cells with human cytomegalovirus (HCMV), a virus that causes ER stress upon infection [29]. Curiously, HCMV evolved molecular strategies to downregulate B7H6 by the viral proteins US18 and US20 [7, 8], which can negate ER stress-mediated induction. A total of 624 wt and PERK KO cells were infected with the HCMV ΔUS17-20 mutant, which lacks the modulators of B7H6. The virus was also equipped with GFP, allowing analysis of the infected cells by flow cytometry. B7H6 surface levels increased in the infected wt, but not in PERK KO cells (Fig. 2), indicating that ER stress at a physiological magnitude induces B7H6 and this is the main mechanism that elevates B7H6 upon HCMV invasion.

### No evidence for translation induction of B7H6 following Tg treatment

**Fig. 3:**
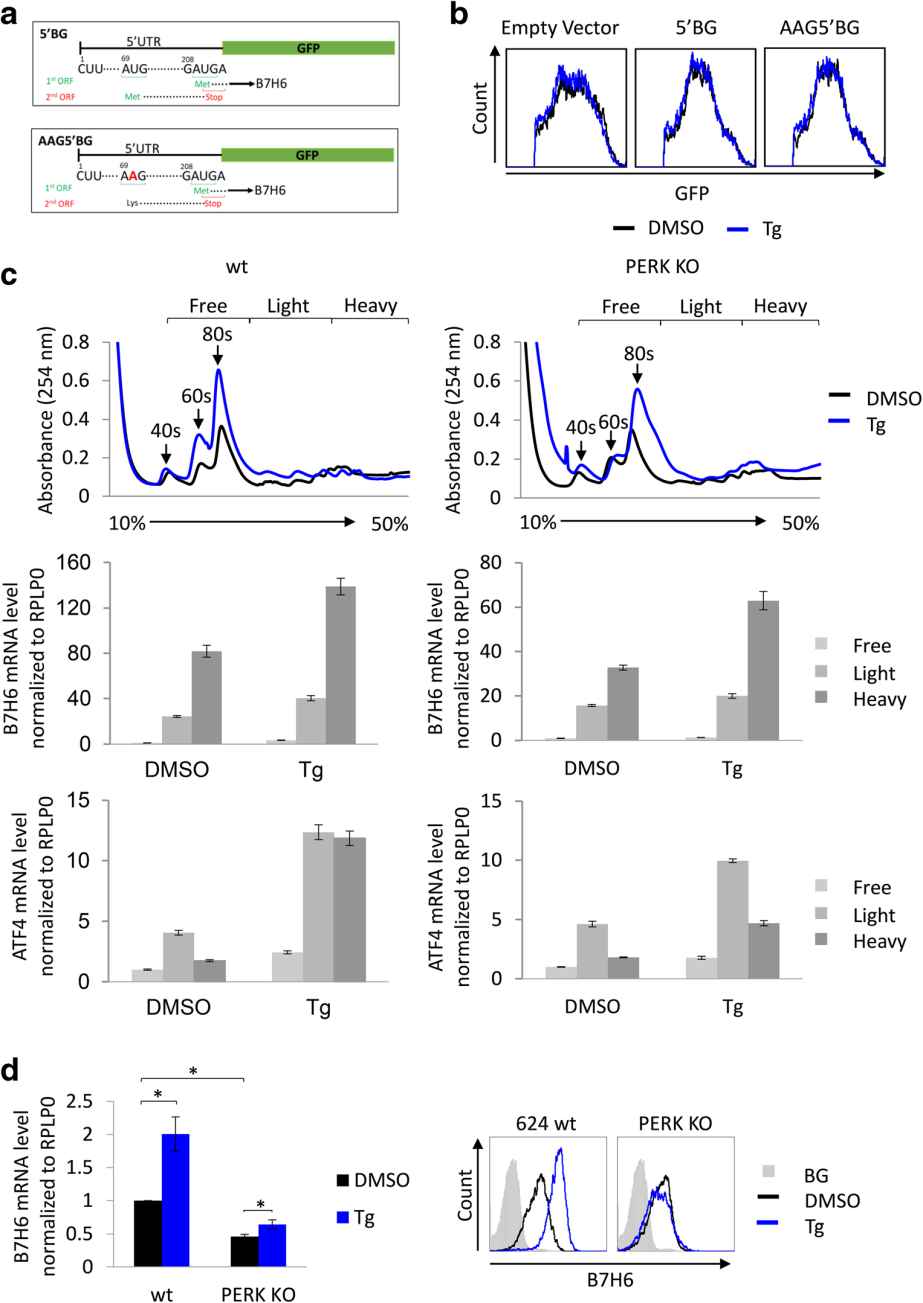
B7H6 induction by ER stress is correlated with its mRNA levels. **a** Schematic representation of the 5′UTR (untranslated region) of B7H6 (5′BG, upper panel), the lower panel shows the mutated nucleotide from U to A, AAG5′BG. **b** Measurement of GFP levels by flow cytometry in 624 wt cells stably expressing empty vector, 5′BG or AAG5′BG constructs after treatment with 0.125 μg/ml Tg or mock treated with DMSO for 16 h. **c** Polysome profiling of B7H6 mRNA in 624 wt or PERK KO cells after treatment with 0.125 μg/ml Tg or mock treated with DMSO for 16 h, presented is the average ± STD of triplicates of the mRNA levels of B7H6 (middle panel) and ATF4 (lower panel) in the different sucrose fractions. **d** Real-time PCR quantification of B7H6 mRNA in 624 wt or PERK KO cells after treatment with 0.125 μg/ml Tg or mock treated with DMSO for 16 h, represented is the average of relative normalized mRNA levels ± STD of three independent experiments. The right panel represents B7H6 surface levels on the tested cells (BG indicates secondary only background staining, shown is the BG for untreated cells)

Phosphorylation of eIF2α induces the expression of proteins, whose mRNA contains a non-productive upstream open reading frame (uORF) [17]. An example is ATF4, whose expression is induced under conditions of hyper eIF2α phosphorylation [18]. Examination of the 5′ untranslated region (5′UTR) of B7H6 identified an uORF in which the upstream start codon ends exactly with a stop codon overlapping with the start codon of the main ORF (Fig. 3a). A similar configuration is in the 5′UTR of the human ATF4 mRNA. To test whether the PERK pathway promotes the translation of B7H6, similarly to that of ATF4, we treated 624 wt cells with guanabenz (Gbz), a molecule suggested to inhibit the inducible phosphatase of eIF2α, growth arrest, and DNA damage-inducible protein (GADD34) [30]. We concluded that Gbz treatment upregulates the B7H6 surface level in both 624 wt and PERK KO cells, as expected. However, we did not observe a significant increase in eIF2α phosphorylation (Fig. S3A and B). Thus, we could not establish a translation regulation control of B7H6 expression.

We decided to append the 5′UTR of B7H6 on the ORF of GFP, forming a translation reporter, termed 5′BG. As a control, we mutated the AUG codon of the uORF, termed AAG5′BG (Fig. 3a). The vectors were expressed in 624 cells. The treatment of cells stably expressing the reporters with Tg demonstrated no changes in fluorescence (Fig. 3b), which suggests the lack of translational control of the B7H6/GFP chimeric mRNA expression under conditions of ER stress. We performed a polysome profiling for B7H6 mRNA in 624 wt or PERK KO cells after Tg treatment to further address the possibility of translation enhancement for B7H6. In contrast to ATF4 mRNA, which partitioned to the heavier fractions under stress conditions, serving as a positive control, no difference was observed in the ratio between the amount of B7H6 mRNA in the heavy and the light polysome fractions after Tg treatment, indicating that any enhancement in translation would be below the detection limits of this analysis (Fig. 3c). However, we observed that Tg treatment increased the total mRNA levels of B7H6.

We, therefore, assumed that the induction of B7H6 expression is due to increase in its mRNA levels. Indeed, the mRNA levels of B7H6 were elevated in wt cells by two-fold following Tg treatment. Total mRNA levels in PERK KO cells were significantly lower than in wt cells and were slightly affected by the treatment (Fig. 3d). This was correlated with the B7H6 surface expression taken from the same samples (Fig. 3d, right panel). These data indicate that induction of B7H6 by ER stress is primarily a transcription response.

The main transcription factor of the PERK pathway is ATF4, induced by combined transcription and translation [18]. ATF4 binds the AARE sequence [31]. A sequence similar to the AARE is deep in the promoter of B7H6 (TGATGCGAA, at − 670 from the transcription start site). However, overexpression of ATF4 in 624 wt cells did not increase B7H6 expression (Fig. S3C), suggesting that the increase in B7H6 mRNA levels under ER stress is most likely independent of ATF4. We generated cells that overexpress B7H6 driven by the lentiviral promoter to further address a possible post-transcription effect of ER stress on B7H6. B7H6 expression was not affected by ER stress in these cells (Fig. S3D). We conclude that ER stress through the PERK pathway enhances the mRNA levels of B7H6 by an undefined mechanism. We have not detected a significant contribution of post-transcription events.

### HIV protease inhibitors induce persistent B7H6 expression

**Fig. 4:**
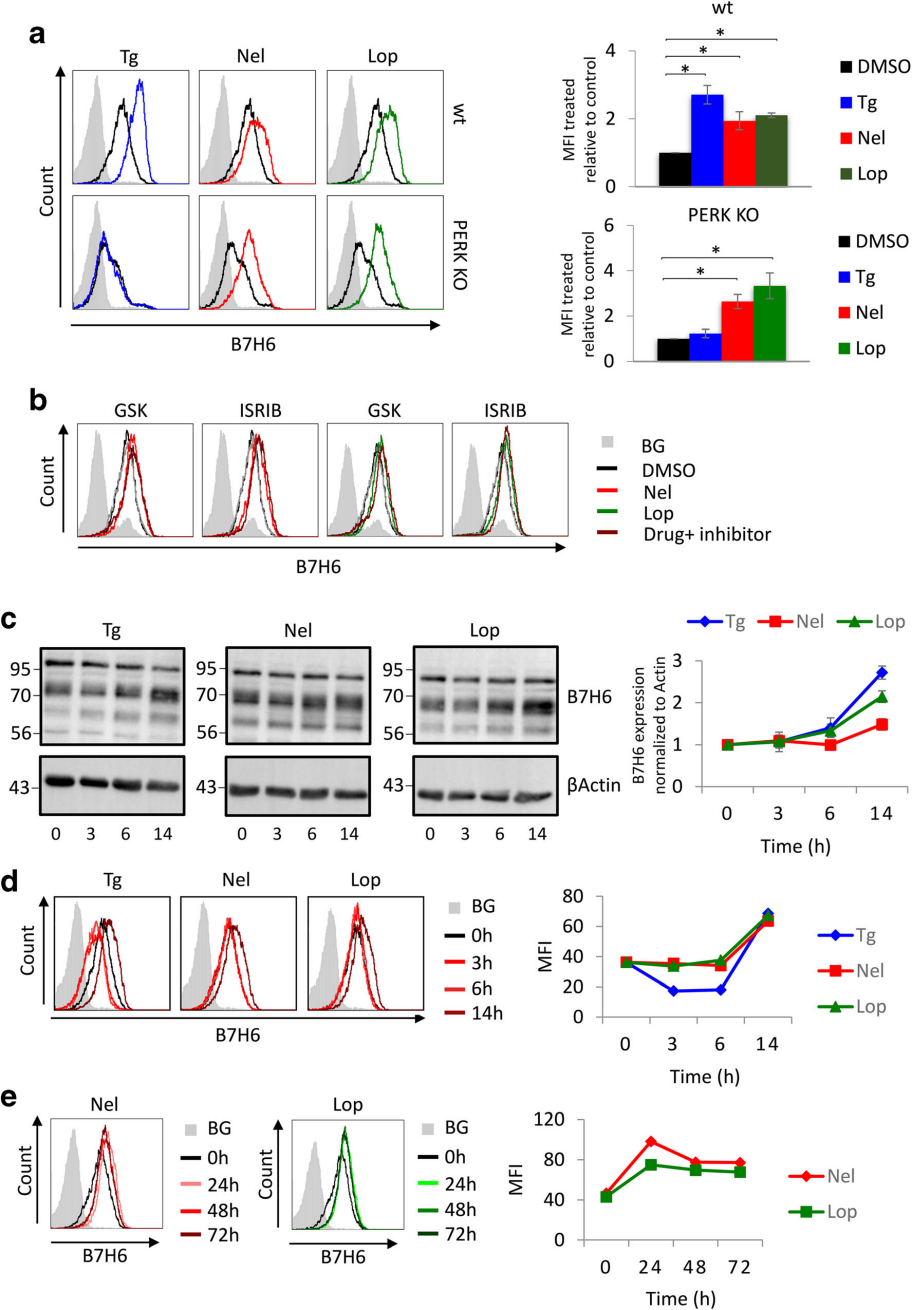
Nelfinavir and Lopinavir sustainably induce B7H6 expression. B7H6 surface levels were evaluated by flow cytometry at the following conditions: **a** after treatment of 624 wt and PERK KO cells with 0.125 μg/ml Tg, 10 μM nelfinavir (Nel), or 20 μM lopinavir (Lop) or mock treated with DMSO for 16 h, to the right appears the average ± STD of the fold change in B7H6 expression of three independent experiments. **b** 624 wt cells pretreated with 1 μM GSK or 0.5 μM ISRIB for 1 h and then with 10 μM Nel or 20 μM Lop or mock treated with DMSO for 16 h. **c** Immunoblotting analysis of B7H6 protein levels in 624 wt cells after treatment with 0.125 μg/ml Tg, 10 μM Nel, or 20 μM Lop for 0, 3, 6, and 14 h, β-actin was used as a loading control. The chart represents quantification of B7H6 normalized to β-actin relative to the zero-time point. **d** B7H6 surface levels on the cells in **c**. **e** Up to 72 h of time-course analysis of 624 wt cells with 10 μM Nel or 20 μM Lop. The charts in **d** and **e** represent the MFI of each time point for each treatment. BG indicates secondary only background staining, which was similar for both treated and untreated cells (shown is the BG for untreated cells)

The induction of B7H6 by ER stress raises the intriguing possibility to promote B7H6 expression by drugs. The HIV protease inhibitors nelfinavir (Nel) and lopinavir (Lop) increase eIF2α phosphorylation by engaging different mechanisms [32, 33]. These anti-viral drugs are taken at daily doses that exceed 1 g and reach a maximal serum concentration of millimolars [34]. Thus, application of these drugs at micromolar concentrations is probably within their pharmacological range. Treatment of 624 wt cells with Tg, Nel, or Lop at low micromolar concentrations significantly induced B7H6 surface levels in a dose-dependent manner (Fig. 4a; Fig. S4A). A positive effect of the drugs was also observed in the acute monocytic leukemia cell line THP-1 (Fig. S4B), indicating the response of hematopoietic tumor cells to ISR induction. Of note, some tumors express B7H6 and some do not. HCT-116 is a colorectal carcinoma cell line which does not express B7H6. Treatment of HCT-116 with Tg, Nel, or Lop did not promote B7H6, suggesting that a basal level of expression is needed for a further induction by the drugs (Fig. S4B).

We also tested the effects of the drugs on PERK KO cells. As noted above, the basal level of B7H6 in PERK KO cells is lower. In distinction to Tg, both drugs were effective also in the PERK KO cells (Fig. 4a), suggesting the involvement of the ISR rather than UPR alone in promoting B7H6 expression. As expected, B7H6 induction following Nel or Lop treatment was not inhibited by the PERK inhibitor. However, ISRIB was also not effective in compromising B7H6 elevation by the HIV protease inhibitors, possibly owing to its low activity (Fig. 4b) [28].

We performed a time course analysis followed by immunoblotting and flow cytometry analyses for B7H6 to characterize the kinetics of B7H6 induction by the different pharmacological agents. B7H6 protein levels started to rise after 6 h of treatment (Fig. 4c) and accumulated at the surface 14 h after the treatment (Fig. 4d). The elevated B7H6 reached a steady state that was maintained throughout the treatment up to 72 h (Fig. 4e). These data show that Nel and Lop are sustainable inducers of B7H6 surface expression at their pharmacological concentration irrespectively of PERK.

### B7H6 upregulation enhances the activity of B7H6-directed CAR-T cells

**Fig. 5:**
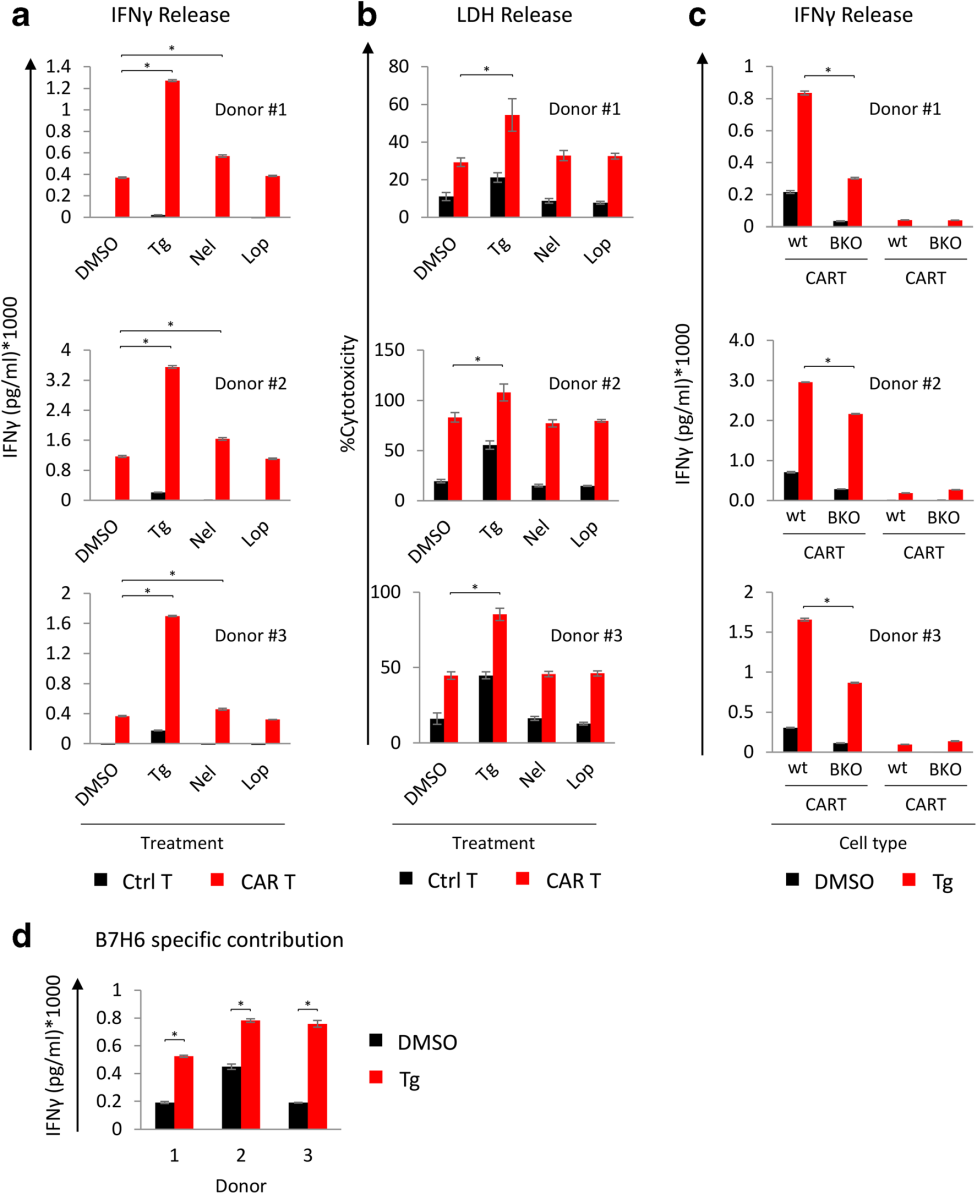
Treatment with Tg or Nelfinavir enhanced the activity of B7H6-directed CAR-T cells. **a** IFNγ concentration (pg/ml) secreted by B7H6 specific CAR T or control T cells after 24 h of co-culture with 624 wt cells pretreated with 0.125 μg/ml Tg, 10 μM Nel, or 20 μM Lop for 16 h. CAR T cells treated with 20 ng/ml PMA and μg/ml ionomycin were used as positive control and CAR T cells that were not cultured with 624 cells were used as negative control (IFNγ levels for the positive and the negative controls are not shown). **b** % specific cytotoxicity for the target cells in **a** calculated by LDH activity as follows: %cytotoxicity = [(treated LDH activity) − (spontaneous LDH activity)]/[(maximum LDH activity) − (spontaneous LDH activity)] * 100. The bars represent the average ± STD of triplicates of CAR T cells from three different donors. **c** IFNγ concentration (pg/ml) secreted by B7H6-specific CAR T or control T cells after 24 h of co-culture with 624 wt or B7H6 KO cells under control or Tg treatment as in **a**. **d** B7H6-specific contribution to the CAR T cell activity. This contribution was calculated by subtracting the IFNγ concentration values of the KO cells from the concentration values of wt cells for DMSO or Tg-treated cells. Statistical significance was determined by Kruskal–Wallis one-way analysis of variance at *p < 0.05

**Fig. 6:**
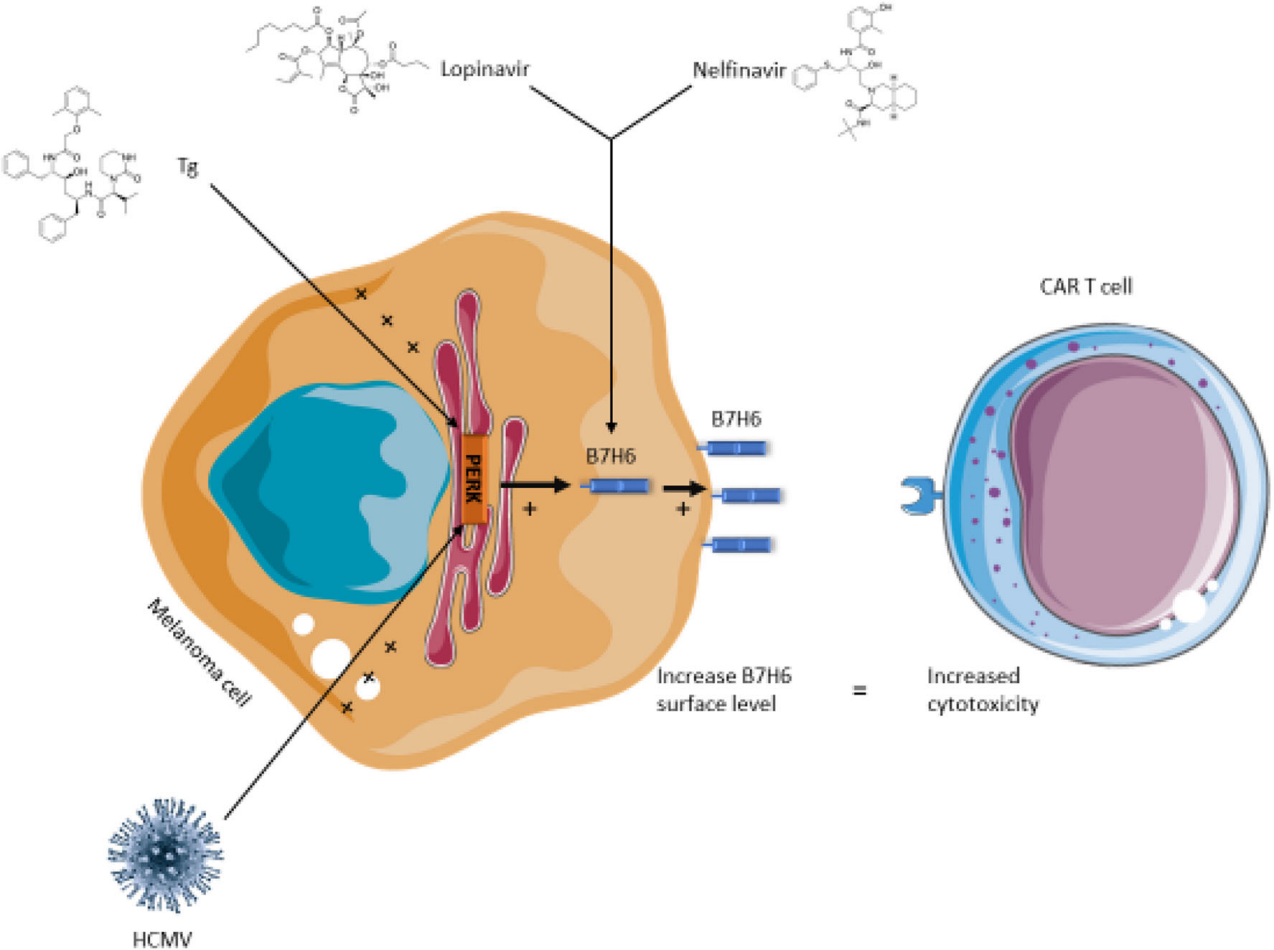
A model for the pharmacological treatments that enhance B7H6 expression and the subsequent enhanced activation of B7H6-specific CAR-T cells

Nelfinavir also shows anti-cancer properties [35, 36] in addition to its anti-viral activity. Several clinical trials have been conducted using nelfinavir in combination with chemotherapy. However, addition of nelfinavir to potentiate cancer immunotherapy has not been analyzed. We utilized engineered T cells with anti-B7H6 CAR from three different donors to test whether the induction of B7H6 by the ER stress inducers could enhance the activity of B7H6-directed CAR-T cells. We measured IFNγ from the T cells and LDH release from treated 624 cells after incubation with non-engineered T cells (background) and the corresponding CAR-T cells. The data show that treatment with Tg and Nel significantly induced IFNγ secretion from all the three donors (by 260% and 40% on average, respectively). Tg treatment also promoted CAR-T-mediated cell cytotoxicity (LDH release). Lopinavir did not significantly improve the CAR-T performance (Fig. 5a, b). These data indicate that Tg, or nelfinavir to a lower extent, improves CAR-T activation and killing. To ensure that the increase in IFNγ release was related to the enhanced B7H6 expression, we utilized the B7H6 KO cells as targets. Interestingly, IFNγ levels increased for all three donors also when encountering Tg-treated B7H6 KO targets (Fig. 5c), suggesting that other factors may play a role. However, the Tg effect was significantly stronger for the B7H6-expressing cells than for the KO controls. We plotted the differences between the two targets for each of the donors (Fig. 5d). Thus, the elevation in B7H6 can fortify the anti-tumor response. A cartoon that summarizes the effects of Tg, Nel, Lop, and HCMV on B7H6 expression and the subsequent combination with CAR-T cells is shown in Fig. 6.

## Discussion

Immunotherapy has revolutionized traditional cancer treatment. Recent advances in using checkpoint inhibitors and adoptive cell therapy as well as clinical success have placed it at the center of attention for therapy development. In this context, melanoma shows a great albeit limited potential, with only 30% of patients responding to the treatment [37]. Understanding the underlying reasons for the lack of response for the rest of the patients has stimulated intense research. Combinations of chemotherapy and immunotherapy increase efficacy [38]. In this study, we have chosen melanoma, which is a primary target of immunotherapy among solid tumors and because B7H6 is expressed in melanoma and not in normal melanocytes [3].

All three arms of the UPR are involved in cancer biology. The PERK pathway in particular is involved in almost all of the steps of cancer biology from initiation to therapy. For instance, PERK is activated and is required for Myc-dependent transformation [39] and the development of resistance to chemotherapy [40]. In melanoma, particularly in the BRAF mutated tumors, PERK is particularly important and serves oncogenic properties indicating its constitutive activation [41]. Here, we present a new function of the PERK pathway that can be exploited for improved immunotherapy.

The PERK arm is responsible for global protein translation inhibition under ER stress. Some cellular proteins, such as ATF4, CHOP, and Bip, escape this inhibition and are induced when PERK is activated [42–44]. The effect of PERK on cellular proteome is more complex, shifting its regulation mechanism under chronic ER stress conditions. This mechanism, involving the translation initiation factor eIF3, affects mRNA molecules without uORF elements [28] and probably expands the translation effects of the UPR as recently was shown in MEFs [19].

B7H6 mRNA was induced by ER stress. However, the magnitude of surface upregulation consistently exceeded the induction in transcript levels (Fig. 3d), suggesting examination of a role for translation. While it is possible for a modest translation enhancement owing to the potential inhibitory uORF in the 5′UTR of B7H6 mRNA, we have not been able to detect any using reporters, polysome analyses, and generating stable infectants with the B7H6 main ORF (Fig. 3; Fig. S3C and D). It will be useful to explore whether the uORF is actually expressed under normal and ER stress conditions [44].

The induction of B7H6 via Tg and under HCMV infection (Figs. 1 and 2) led us to think that clinically approved drugs that induce mild stress conditions may promote B7H6 expression. HIV protease inhibitors known to induce ER stress, nelfinavir and lopinavir, were able to induce a sustained B7H6 surface levels on 624 cells (Fig. 4). In contrast to Tg, both nelfinavir and lopinavir induced B7H6 also in PERK-deficient cells (Fig. 4). While for nelfinavir it is likely that the direct inhibition of the eIF2α phosphatase PPP1R15B plays a role in this effect [32], the mechanisms by which lopinavir instigate ER stress are less understood [45]. In our opinion, this serves as an advantage for use as an inducer of B7H6 independently of PERK activation. B7H6 was reported to be induced by a number of diverse treatments, for which a common mechanism was not proposed. This includes the chemotherapeutic drugs cisplatin and 5-FU, radiation and heat shock, and other stressful conditions [4]. Cisplatin was shown to induce phosphorylation of eIF2α [46] and so does heat shock [47]. The connection between ionizing radiation and phosphorylation of eIF2α is not well established. It is tempting to speculate that ISR may be a common denominator for several of the treatments that induce B7-H6. This deserves further investigations.

An additional advantage to combine nelfinavir with B7H6-directed immunotherapy is the anti-cancer effects of nelfinavir as a standalone therapy [35] or in combination with other drugs [36]. As proof of this concept, we examined CAR-T activation in the presence and absence of ER stress inducers. We showed that pretreatment of 624 cells with Tg or nelfinavir significantly induces the activation of B7H6-specific CAR-T cells (Fig. 5). The elevation in IFNγ release was not entirely attributed to the presence of B7H6, as shown also for B7H6-deficient targets. This can be a result of danger signals released from dying cells following Tg treatment or even a residual effect of Tg leftovers on the T cells. However, a contribution for B7H6 induction was demonstrated, which was augmented under Tg treatment (Fig. 5d). Although lopinavir induced B7H6 as nelfinavir, it did not enhance the activity of B7H6-specific CAR-T cells. While we have not investigated the underlying reasons in detail, it can be due to effects on other immune recognition molecules by lopinavir. Although LDH release significantly increased with Tg treatment, treatment with nelfinavir or lopinavir did not show significant increase, apparently due to the initial high lysis compared to the untreated cells owing to the potency of the CAR-T cells. It should be noted that while CAR-T is highly efficient for the treatment of B cell malignancies [48, 49], resistance in solid tumor therapy has been documented [50]. The addition of drugs that will minimize the resistance is highly pursued. We propose that incorporation of HIV protease inhibitors may have a significant effect on the efficacy and development of resistance for B7H6-targeted therapy. To the best of our knowledge, this pharmacological approach has not yet been tested.

## Electronic supplementary material

ESM 1 (PDF 1350 kb)
