# Supplementary material for: The integrated stress response promotes B7H6 expression
Source: J Mol Med (Berl). 2019 Dec 14;98(1):135–48. doi: 10.1007/s00109-019-01859-w (PMC6952340; doi:10.1007/s00109-019-01859-w)
Supplement: Supplementary file 1 — (PDF 1350 kb) [file 109_2019_1859_MOESM1_ESM.pdf]

A

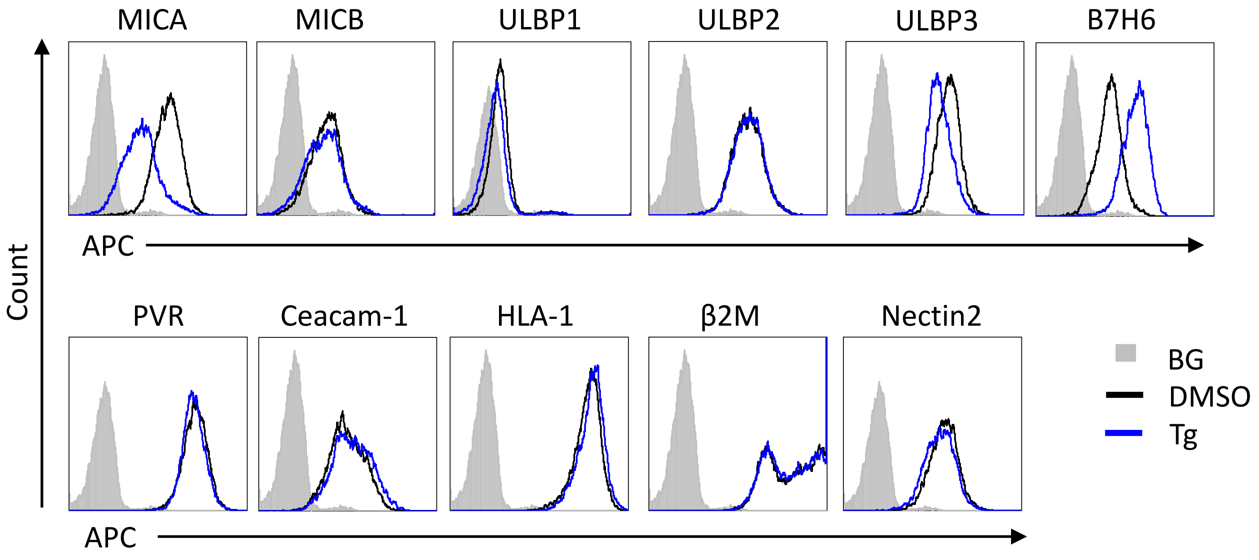

Fig S1. Effect of ER stress on the expression of NK cells' ligands. **(A)** The expression of the indicated natural killer cells ligands was tested by flow cytometry on 624 wt cells treated with 0.125 µg/ml Tg or mock treated with DMSO for 16 hours. BG indicates secondary only background staining for both treated and untreated cells (shown in silver is the BG for untreated cells).

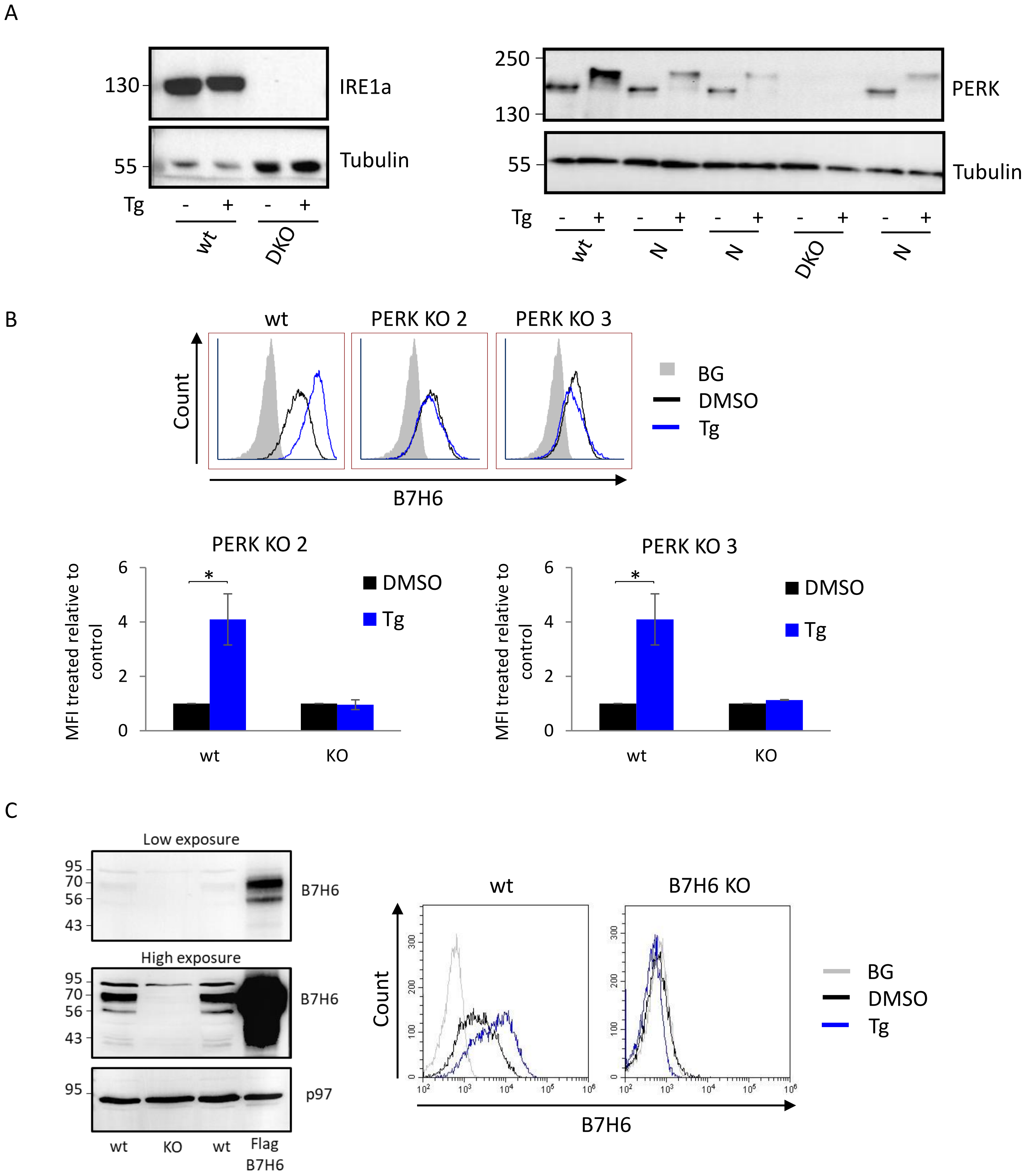
A
130
IRE1a
55
Tubulin
Tg
- + - +
wt
DKO
250
130
PERK
55
Tubulin
Tg
- + - + - + - + - +
wt
N
N
DKO
N
B
wt
PERK KO 2
PERK KO 3
Count
B7H6
BG
DMSO
Tg
PERK KO 2
MFI treated relative to control
6
4
2
0
*
DMSO
Tg
wt
KO
PERK KO 3
MFI treated relative to control
6
4
2
0
*
DMSO
Tg
wt
KO
C
Low exposure
95
70
56
43
B7H6
High exposure
95
70
56
43
B7H6
95
p97
wt
KO
wt
Flag B7H6
wt
B7H6 KO
Count
B7H6
BG
DMSO
Tg

Figure S2

Fig S2. Generation of 624 knockout cells. **(A)** Immunoblotting analysis of IRE1 (left) and PERK (right) protein levels in 624 double knockout (DKO) for both genes after treatment with 0.125 µg/ml Tg or mock treated with DMSO for 16 hours. Tubulin was used as a loading control. N indicates negative knockout clones. **(B)** Analysis of B7H6 surface levels on another two clones of 624 PERK KO cells after treatment with 0.125 µg/ml Tg or DMSO for 16 hours compared to wt cells. The charts to the right shows the average mean fluorescence intensity (MFI) ± STD of treated relative to untreated cells of three independent experiments. **(C)** Immunoblotting analysis of B7H6 protein levels in 624 wt or B7H6 KO cells, p97 was used as a loading control. The right panel shows B7H6 surface levels as assessed by flow cytometry on those cells after treatment with 0.125 µg/ml Tg or DMSO for 16 hours. BG indicates secondary only background staining for both treated and untreated cells (shown in silver is the BG for untreated cells).

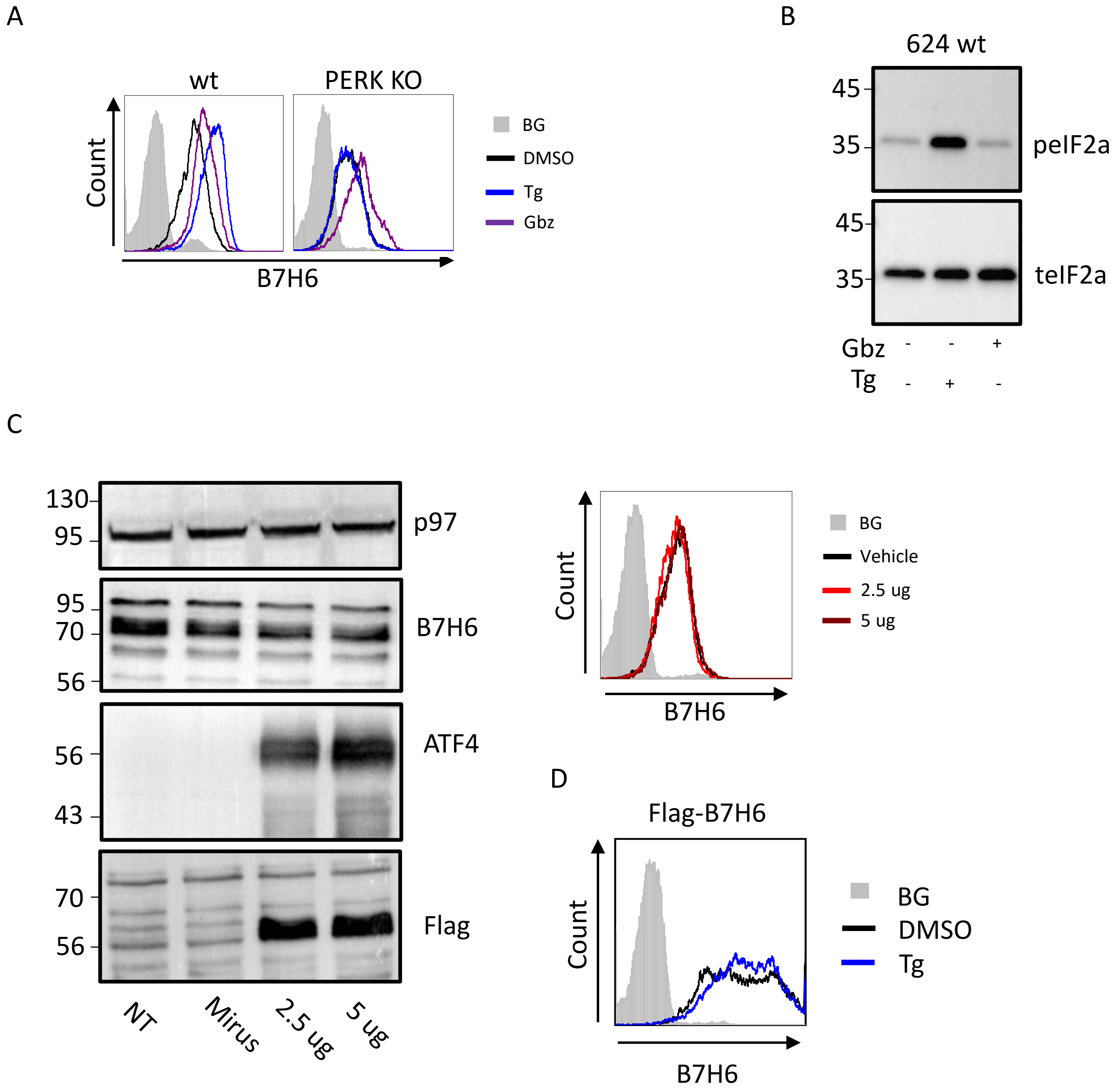

Figure S3. Guanabenz and ATF4 effects on B7H6 expression. **(A)** B7H6 surface levels were evaluated by flow cytometry on 624 wt and PERK KO cells treated with 0.125 µg/ml Tg or 50 µM guanabenz (Gbz) or mock treated with DMSO for 16 hours. **(B)** Immunoblotting analysis for peIF2α and total eIF2α in the cells in A. **(C)** Immunoblotting analysis of B7H6, ATF4 and Flag tag levels in 624 wt cells transfected with 2.5 or 5 µg of Flag-ATF4. p97 was used as a loading control. To the right appears B7H6 surface levels evaluated by flow cytometry on the transfected cells. **(D)** 624 wt stably expressing Flag-B7H6 were treated with 0.125 µg/ml Tg or mock treated with DMSO for 16 hours. Then, they were evaluated for B7H6 expression by flow cytometry. BG indicates secondary only background staining for both treated and untreated cells (shown is the BG for untreated cells).

A

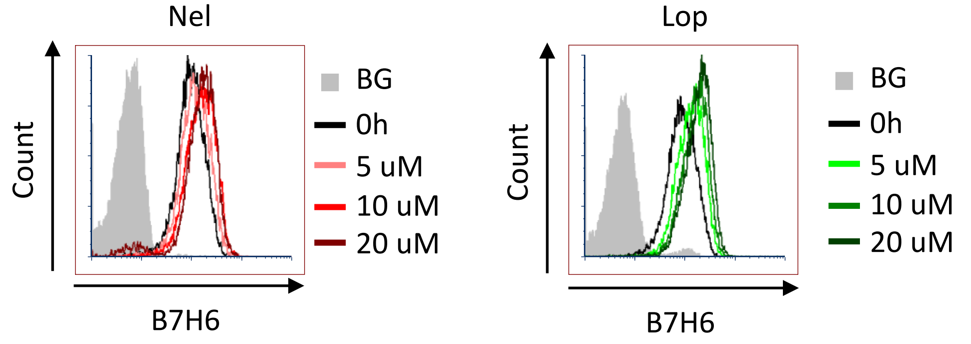

B

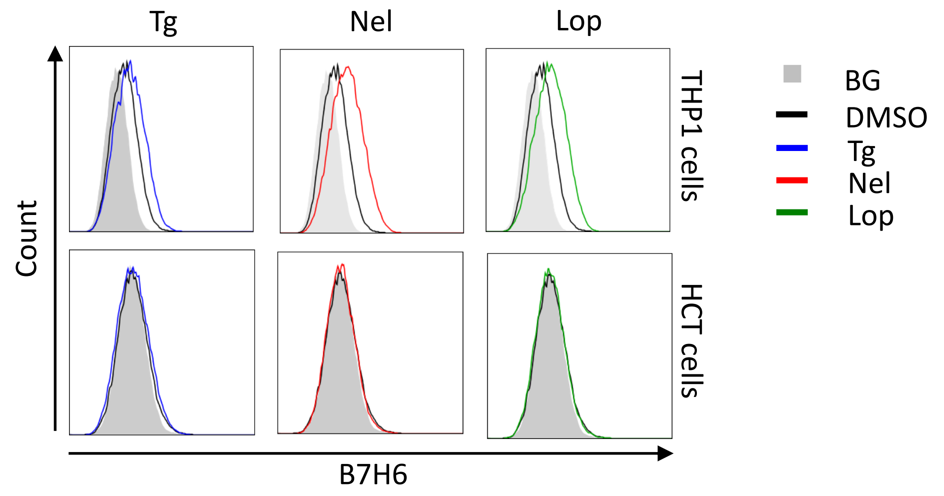

Figure S4. Nelfinavir and lopinavir dose response and their effect on THP1 or HCT cell lines. **(A)** 624 wt cells were treated with the indicated concentrations of each drug for 16 hours or mock treated with DMSO. Then, B7H6 surface levels were evaluated by flow cytometry. **(B)** B7H6 surface level were evaluated by flow cytometry on THP1 or HCT cells after treatment with 0.125 µg/ml Tg, 10 µM Nel or 20 µM Lop or mock treated with DMSO for 16 hours. BG indicates secondary only background staining, which was similar for both treated and untreated cells (shown is the BG for untreated cells).
